# Stone Matrix Asphalt with Fischer–Tropsch Wax and Recycled Rubber: A Multi-Scale Evaluation of Mechanical and Functional Performance

**DOI:** 10.3390/ma19050928

**Published:** 2026-02-28

**Authors:** Roman Pacholak, Biruh Alemayehu Seyoum, Mohamed Eladly

**Affiliations:** Department of Geotechnics, Roads and Geodesy, Faculty of Civil Engineering and Environmental Sciences, Bialystok University of Technology, 15-351 Bialystok, Poland; biruhalemayehuseyoum@gmail.com (B.A.S.); eladly_m@icloud.com (M.E.)

**Keywords:** crumb rubber, Fischer–Tropsch wax, warm mix asphalt, sustainable infrastructure, pavement performance

## Abstract

**Highlights:**

**What are the main findings?**

- The 4% FTW achieves an optimal balance between rutting resistance and aging durability in PMB.
- The FTW–RP synergy reduces the thermal cracking failure temperature by up to 8.0 °C.
- The 4% FTW enables a 20 °C reduction in production temperature for SMA11 mixtures.
- The 15% RP promotes self-texturing and reduces braking distance by 13% after accelerated polishing.
- RP dosage governs performance outcomes: 5% for moisture resistance, 10% for noise reduction, 15% for mechanical durability.

**What are the implications of the main findings?**

- Hybrid modification supports sustainable road construction through waste tire reuse and literature-supported emission reductions associated with lower production temperatures.
- A multi-scale approach links binder rheology directly to long-term pavement functionality.
- The results enable targeted tailoring of asphalt properties to meet specific urban or heavy-duty pavement requirements.
- Self-texturing rubberized surfaces significantly enhance long-term road safety.

**Abstract:**

This study investigates the synergistic use of Fischer–Tropsch wax (FTW) and recycled rubber powder (RP) as dual modifiers in stone mastic asphalt (SMA11) to improve its mechanical and functional performance. Rheological analysis demonstrated that an FTW content of 4% achieves the optimal balance of high-temperature rutting resistance, aging resistance, and workability, with a binder viscosity of 1.6 Pa·s at 135 °C. When incorporated into SMA11 mixtures at 15%, RP yielded the best overall mechanical performance, including a reduction in rut depth to 1.22 mm and a 25% decrease in wheel tracking slope (WTS). The 15% RP mixtures also exhibited superior long-term skid resistance (μ_m_ = 0.329 after 180,000 polishing cycles, corresponding to a 13% reduction in braking distance) and enhanced thermal cracking resistance (failure temperature improved by 8.0 °C to −32.7 °C). An RP content of 5% maximized moisture resistance (ITSR = 100%), while 10% RP produced the highest mid-frequency sound absorption coefficient (α = 0.050). The hybrid modification system enables a 20 °C reduction in production temperature, consistent with published data on wax-based warm-mix technologies, and is associated with reduced energy consumption and lower emissions. The approach simultaneously supports sustainable pavement design through the high-value reuse of waste tire rubber.

## 1. Introduction

Asphalt concrete has long been the dominant material for road construction owing to its combination of strength, durability, and cost-effectiveness [1]. Despite constituting only a minor fraction of the total mixture by mass, the bituminous binder is the component that governs long-term pavement performance. Its colloidal and viscoelastic nature renders it highly sensitive to temperature fluctuations and repeated loading, such that even minor changes in binder composition can precipitate rutting, cracking, and premature aging [2,3,4]. Conventional petroleum-derived binders progressively stiffen and oxidize during service, thereby reducing pavement life and increasing maintenance demands [5,6]. These limitations have motivated extensive research into chemical and physical binder modification strategies aimed at enhancing both durability and rheological performance [7,8,9].

The incorporation of crumb rubber (CR) derived from end-of-life tires has emerged as one of the most promising and sustainable binder modification approaches [10,11,12]. CR modification improves binder flexibility, fatigue resistance, and crack healing capacity, while simultaneously advancing circular economy objectives through the valorisation of waste tire material [13,14,15,16]. Numerous studies have confirmed that CR reduces permanent deformation susceptibility and lowers pavement noise levels [6,16,17,18]. However, the incorporation of rubber particles substantially increases binder viscosity, necessitating elevated mixing and compaction temperatures in the range of 170–190 °C [19,20]. These high production temperatures accelerate binder oxidation, increase energy consumption, promote rubber network degradation, and generate excessive pollutant emissions, thereby partially offsetting the environmental benefits of waste rubber reuse [21,22,23]. Reducing production temperatures without sacrificing mechanical performance has therefore become a key research priority [24,25]. Warm mix asphalt (WMA) technologies represent a significant advance in this regard [26,27,28], enabling mixing and compaction at temperatures 20–40 °C below those required for conventional hot mix asphalt (HMA) without compromising structural integrity or durability [29]. The resulting reductions in fuel consumption and CO_2_ emissions make WMA an environmentally attractive alternative [30,31,32]. Lower processing temperatures also facilitate the increased use of reclaimed asphalt pavement and other recyclable materials, and mitigate binder aging during production [33,34]. FTW is a synthetic, long-chain aliphatic hydrocarbon produced from natural gas or biomass via the Fischer–Tropsch process. It has attracted considerable interest as a WMA additive owing to its ability to reduce binder viscosity and improve mixture workability during production and compaction [25,35,36]. When incorporated at dosages of 1–4% by binder mass, FTW enhances aggregate coating, compaction efficiency, and rutting resistance through crystallisation-induced stiffening at service temperatures [37,38,39,40]. Ameri et al. [6] reported that the addition of 2% FTW to a binder containing 15% CRM increased elastic recovery by 46%, although it also reduced viscosity and creep recovery. Moderate FTW dosages have been shown to enhance both mechanical performance and production efficiency [41,42]; however, excessive wax content can embrittle the binder and reduce low-temperature flexibility [43,44,45,46]. Desidery and Lanotte [10] noted that FTW contents of 4–6% increase stiffness at the expense of elasticity, while Raihan et al. [11] observed a reduction in plasticity above 4% wax content. Identifying the optimal FTW dosage that maintains a satisfactory balance between rutting resistance and fatigue performance therefore remains a critical design challenge [3,47].

The combined use of CR and FTW represents a promising dual-modification strategy for eco-efficient asphalt pavements. In this approach, CR enhances binder flexibility and mixture durability, while FTW counteracts the viscosity increase associated with rubber incorporation, facilitating production and compaction at reduced temperatures [48,49,50,51]. The synergistic interaction between the two modifiers yields a binder that is more resistant to permanent deformation and less susceptible to flow at elevated temperatures compared with either modifier used alone [16,41]. Yang et al. [16] demonstrated that HMA incorporating both CR and FTW additives reduced production temperature by approximately 27 °C and decreased toluene and benzene emissions by approximately 30%. The environmental benefits of this combined approach, including reductions in fuel consumption and greenhouse gas emissions, have been further corroborated by Yildirim et al. [17] and Sukhija et al. [4].

Despite these promising results, the mechanistic interactions between CRM and FTW remain insufficiently understood. The majority of published studies have focused narrowly on binder-scale rheological parameters, including complex shear modulus (|G*|), phase angle (δ), viscosity, and non-recoverable creep compliance J_nr_ [18,19,30,42], while neglecting mixture-level properties of direct practical relevance, such as stiffness, moisture resistance, sound absorption, and skid resistance. The limited mixture-level data available reveal conflicting trends: Gui et al. [18] and Kataware and Singh [19] reported that CR-modified binders processed at elevated temperatures deliver excellent rutting resistance and energy efficiency, yet may exhibit reduced fatigue life at high wax concentrations or following prolonged aging. Furthermore, at reduced production temperatures, FTW may impede the activation and swelling of rubber particles, thereby diminishing the degree of binder modification [6,10,11,31]. This mechanistic complexity accounts for the divergent outcomes reported in the literature, which range from enhanced synergy [22,24,32] to neutral or adverse interactions [33,37,38]. Research specifically addressing the technical performance of asphalt mixtures containing FTW and CR processed via the ‘dry’ blending method is particularly scarce.

Environmental considerations further complicate the picture. Compared with unmodified HMA, FTW-modified mixtures can release significantly elevated concentrations of volatile organic compounds (VOCs), including toluene, xylene, and benzene, with emissions reported to be up to six times higher than those from unmodified HMA [16,25,26]. Conversely, the lower processing temperatures and reduced fuel demand characteristic of FTW-based WMA substantially mitigate these emissions [16,17,27]. In addition, synthetic waxes such as FTW enhance compaction efficiency in cold or high-altitude environments where conventional HMA mixtures lose workability too rapidly [7,13,20]. Taken together, the combined use of CRM and FTW constitutes an environmentally sound strategy aligned with global sustainability objectives and circular economy principles [28,29,34]. The push for sustainable infrastructure has led to a surge in ‘dual-pathway’ utilization of industrial by-products. For instance, recent research on coal gangue concrete emphasizes a dual approach through aggregate substitution and cementitious activity activation to optimize structural performance [52,53]. This trend of high-value waste integration is mirrored in the asphalt industry, where RP is used not only as a filler but as an active elastomeric modifier to enhance binder properties. Furthermore, understanding the durability of these complex systems requires a deep dive into failure mechanics. Just as recent studies on coal failure under dynamic loading utilize energy evolution mechanisms and fractal characteristics to predict material breakdown, the evaluation of SMA mixtures must account for the dissipation of energy within the hybrid mastic to prevent premature rutting and cracking. Integrating these diverse perspectives allows for a more holistic design of resilient, low-emission pavement structures.

Significant knowledge gaps nevertheless persist. Reported optimal FTW dosages remain inconsistent across the literature [11,48]; some studies advocate higher wax contents [30,36,43], while others [14,18,19,21] suggest that 2% FTW combined with 15% CRM achieves the most favourable balance between viscosity reduction and elasticity retention. This lack of consensus underscores the need for rigorous multi-scale investigations that integrate mixture-level functionality and durability with binder rheological characterisation.

To resolve these inconsistencies and bridge the gap between binder-scale characterisation and field-level performance, this study adopts a rigorous multi-scale methodology. Mixture-level performance cannot be reliably inferred from binder rheology alone, as emergent properties arise from complex interactions among aggregates, modifiers, and the mineral skeleton. Accordingly, this approach establishes a direct link between targeted binder optimisation and a comprehensive battery of mechanical and functional tests conducted at the mixture level. This enables a rigorous assessment of the true contribution of each modifier to composite material behaviour, and allows effects attributable to straightforward mixture design changes (such as partial filler substitution by RP) to be distinguished from those arising from genuine material-level interactions among FTW, RP, and the existing SBS polymer network. By linking the binder and mixture scales, the study provides a more reliable framework for evaluating the influence of dual modification on the long-term durability and performance of SMA mixtures. The present study focuses on experimental performance screening and the mechanistic interpretation of FTW and RP interactions. In parallel, recent advances in computational mechanics, including high fidelity finite element modelling, multi-scale homogenisation, and multi-disciplinary optimisation of complex hybrid structures, underscore the potential of virtual prototyping and dynamic simulation for the assessment of asphalt mixtures. Examples include carbon fibre reinforced sandwich panels and aluminium honeycomb railway carbodies [54,55]. These approaches offer promising directions for future research aimed at simulating internal stress distributions, optimising layer configurations, and predicting long-term field behaviour under realistic traffic and environmental loading conditions.

Building on the critical knowledge gaps identified above, this study pursues three specific objectives: (1) to determine the optimal FTW dosage within a PMB binder co-modified with RP; (2) to quantify the multi-functional performance benefits of the combined FTW+RP system in SMA mixtures across rutting, thermal cracking, moisture resistance, skid resistance, and acoustic properties; and (3) to provide a mechanistic interpretation of the observed effects by establishing correlations between binder-scale rheological changes and mixture-scale performance outcomes. In doing so, this work moves beyond the often contradictory findings in the existing literature to offer a validated, performance-driven framework for the design of sustainable asphalt pavements incorporating dual modifiers.

Although a full multi-factor experimental design (e.g., response surface methodology or orthogonal array) would permit even finer simultaneous optimisation of FTW and RP contents, the phased approach adopted here, comprising binder-level FTW optimisation followed by mixture-level RP variation, yields clear, statistically supported guidelines for tailoring SMA11 mixtures to specific field requirements and represents a sound and practical first step towards sustainable hybrid modification.

## 2. Materials and Methods

### 2.1. Materials

#### 2.1.1. Control Mixture

**Figure 1:**
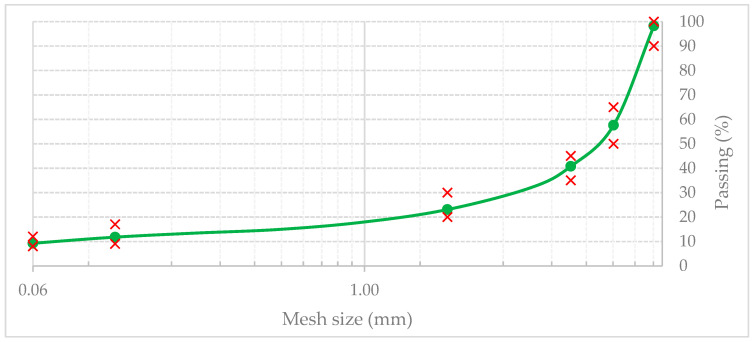
Particle size distribution of SMA11 mixture. 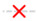
—upper and lower passing limits on each individual sieve.

The reference mixture was stone mastic asphalt with a maximum aggregate size of 11 mm (SMA11), a well-established pavement type recognized globally for its high-performance wearing course and excellent rutting resistance. As a result, any improvement in its functional and mechanical properties is of direct practical relevance to modern road engineering and pavement design [56]. The particle size distribution of the aggregate is shown in Figure 1.

The aggregates and PMB 45/80–55 binder met the quality requirements specified in EN 13108–5 [57]. The aggregate mixture was sourced from a local quarry in the Lower Silesia region of Poland. Finely ground limestone powder was used as the mineral filler, in accordance with EN 13043 [58]. The PMB 45/80–55 was a commercially available polymer-modified bitumen characterized as an elastomeric SBS (styrene–butadiene–styrene) polymer-modified binder. As one of the most commonly used polymer-modified binders in Europe, it represents a suitable base for a wide range of high-traffic pavement applications. The basic properties of the aggregates are summarized in Table 1, and Table 2 presents the viscoelastic properties of the base binder.

#### 2.1.2. Modifiers

The wax used in this study was a high-purity synthetic material supplied by a European manufacturer, characterized by a narrow carbon chain length distribution and a fine crystal structure. It is a white, fine-grained powder with a melting point of approximately 108–114 °C, which is slightly higher than that of traditional wax additives. These properties make it particularly effective for modifying polymer-modified bitumen, as it enhances thermal stability at high temperatures without compromising low-temperature performance. The key properties of FTW are summarized in Table 3. Three wax contents 2%, 4%, and 6%, were evaluated to determine the optimum dosage for modifying PMB 45/80–55 bitumen.

**Figure 2:**
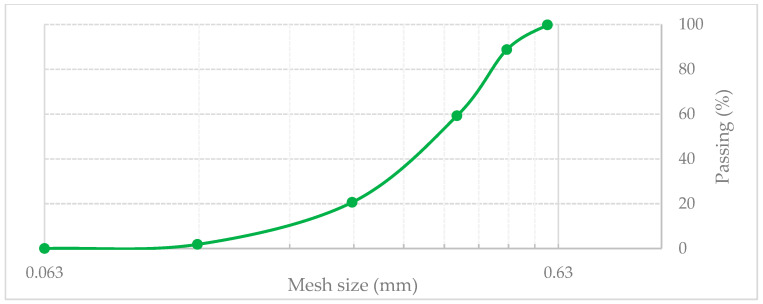
The particle size distribution of the used recycled RP.

The recycled rubber powder (RP) used in this study was a commercial product obtained by ambient temperature grinding of end-of-life tires. A fraction with a maximum particle size of 0.6 mm was selected. Prior to incorporation into the asphalt mixtures via the dry method, the RP was preheated to 60 °C for 30 min to ensure adequate dryness and to promote its interaction with the bituminous binder. The main properties of the recycled RP are presented in Table 4, while its particle size distribution is shown in Figure 2.

### 2.2. Methods

#### 2.2.1. Mixture Production and Compaction Protocol

Modification of PMB 45/80–55 with FTW was performed as follows: (i) the base PMB was heated to 180 °C until fully liquefied; (ii) FTW was added gradually at contents of 2%, 4%, and 6% by weight of the binder; (iii) the blend was stirred using a high-shear mixer (Silverson Machines Ltd., Chesham, UK) at 180 °C and 1000 rpm for 10 min to achieve uniform and stable dispersion of the wax in the binder matrix. The blending parameters (temperature, shear rate, and mixing time) were established through preliminary laboratory tests to ensure complete dissolution and uniform dispersion of FTW within the bituminous matrix. This resulted in a stable modified binder with no signs of phase separation.

Four different SMA11 mixtures were designed and produced. The base binder for all mixtures was PMB 45/80–55 modified with the optimum content of 4% FTW. The selection of a 4% FTW dosage as a constant for the mixture-level study was driven by binder-scale results showing it to be the technical optimum. Exceeding this dosage (6%) led to a significant loss of plasticity (*p* = 0.027), while lower dosages (2%) provided insufficient workability benefits. By maintaining binder modification at this optimal level, the specific contributions of varying RP dosages within the hybrid FTW–PMB matrix could be isolated and quantified. RP was incorporated using the dry method at four proportions: 0% (reference), 5%, 10%, and 15% by weight of binder, replacing an equivalent mass of limestone filler. The optimal binder content was found to be 6.3% of the total mixture weight.

Aggregates were heated to 170 °C. The modified binder, maintained at 150 °C, was added to the heated aggregates and mixed for 180 s in a laboratory mixer (Silverson Machines Ltd., Chesham, UK). Based on the compaction analysis, the compaction temperature was successfully reduced from a conventional 145 °C to 125 °C for the FTW-modified mixtures, exploiting the viscosity-reducing effect of the wax. The detailed compaction analysis is presented in Section 3. To evaluate the mechanical and functional performance of the SMA mixtures across multiple scales, three different compaction methods were used to produce specimens with the required geometries. Cylindrical specimens were compacted using a Marshall impact compactor (Infratest Prüftechnik GmbH, Brackenheim, Germany ) (50 blows per side) and were primarily used for volumetric characterization and moisture susceptibility testing. To better simulate field conditions, large asphalt slabs (400 × 300 × 40 mm) were produced with a laboratory roller compactor (CONTROLS S.p.A., Liscate, Italy). From these slabs, prismatic beams were cut for four-point bending and TSRST testing. In addition, circular specimens (∅225 mm) for skid resistance and (∅100 mm) for acoustic absorption were cored from the compacted slabs. Bulk density (ρ_b_) and air void content (V_m_) were calculated according to EN 12697–6 [80] and EN 12697–8 [81].

The mechanical performance of SMA11 mixtures is highly sensitive to air void content; therefore, to ensure a fair comparison between the reference and modified mixtures, the compaction effort was systematically adjusted according to the FTW dosage. The reference mixture (0% FTW), compacted at 145 °C, achieved an air void content of 3.3%. The optimized mixtures containing 4% FTW reached a comparable air void content (V_m_) value of 3.3% (standard deviation 0.3%), even though they were compacted at a significantly lower temperature of 125 °C. This volumetric alignment is essential, as it ensures that the observed improvements in performance characteristics including rutting resistance, durability, and moisture damage resistance can be attributed to the effects of the FTW and RP modifiers rather than to differences in mixture density or compaction level.

#### 2.2.2. Bituminous Binder Characterization

The physical and rheological properties of the bituminous binders were assessed using a comprehensive set of standardized laboratory tests. Binder consistency was assessed by needle penetration at 25 °C in accordance with [65]; high-temperature properties were determined using the softening point test (Ring and Ball method) per [66]; and elastic recovery at 25 °C was measured per [69] to characterize the elastomeric integrity of the polymer-modified binder.

Temperature susceptibility of the binders was quantified using the Penetration Index (PI), calculated from the penetration and softening point values. Dynamic viscosity was measured at 90 °C, 115 °C, and 135 °C using a rotational viscometer in accordance with [68], enabling assessment of mixture workability and the effect of the wax melting transition on binder flow behavior.

Short-term oxidative aging was simulated using the Rolling Thin Film Oven Test (RTFOT) (Infratest Prüftechnik GmbH, Brackenheim, Germany) to evaluate the binder’s susceptibility to oxidative hardening during production and paving. High-temperature rheological behavior and rutting resistance potential were then evaluated using a Dynamic Shear Rheometer (DSR) (Bohlin Instruments Ltd., Cirencester, UK) through determination of the rutting parameter (|G*|/sin δ) over a temperature range from 46 to 82 °C.

#### 2.2.3. Comprehensive Laboratory Performance Testing

Cantabro Loss Test

The Cantabro test was conducted to assess the resistance of SMA11 mixtures to abrasion and raveling. For each mixture variant, three Marshall specimens were prepared with the appropriate binder and target air void content. The tests were carried out on dry specimens conditioned at 25 °C, in accordance with AASHTO T 96 [82]. Each specimen was placed separately in a Los Angeles abrasion machine (CONTROLS S.p.A., Liscate, Italy) without steel balls, and the drum was rotated 300 times. The initial (m_1_) and final (m_2_) mass of each specimen was recorded before and after the test. The Cantabro loss (CL), representing the percentage of material loss due to abrasion, is calculated using Equation (1):

(1) $$CL=\frac{m_{1}-m_{2}}{m_{1}}\times 100\%$$

The mean CL value obtained from the three specimens was reported as the representative result for each SMA11 mixture.

Moisture Susceptibility

Moisture susceptibility of SMA11 mixtures was evaluated using the indirect tensile strength ratio (ITSR) method. For each mixture, six Marshall specimens were prepared and divided into two groups: three unconditioned (dry) and three conditioned (wet). Conditioning followed AASHTO T 283 [83], involving partial saturation (70–80%), freezing at −18 °C for 16 h, and thawing in a 60 °C water bath for 24 h. After conditioning, all specimens were stabilized at 25 °C.

The ITS test was performed by applying a monotonic compressive force along the vertical diameter of each specimen at a constant loading rate of 50 mm/min. The maximum failure load was recorded for each specimen. The indirect tensile strength (ITS) was determined using Equation (2):

(2) $$ITS=\frac{2\cdot P_{max}}{\pi \cdot d\cdot h}$$

where

$P_{max}$—the maximum load (N);

d—the specimen diameter (mm);

h—the specimen height (mm).

ITSR was calculated as the ratio of the average tensile strength of conditioned specimens to that of unconditioned specimens (Equation (3)):

(3) $$ITSR=\frac{{ITS}_{wet}}{{ITS}_{dry}}\times 100\%$$

A higher ITSR value indicates greater resistance of the asphalt mixture to moisture-induced damage.

Rutting Resistance

Rutting resistance was assessed using a Double Wheel Tracker (CONTROLS S.p.A., Liscate, Italy) in accordance with EN 12697–22 [84]. For each mixture variant, two slab specimens (400 × 300 × 40 mm) were conditioned at 60 °C for 6 h before testing. A load of 700 N per wheel was applied for 10,000 cycles at 26.5 cycles/min. The rut depth was recorded continuously throughout the test.

The Wheel Tracking Slope (WTS) and the Proportional Rut Depth (PRD) were determined using Equations (4) and (5), where RDx denotes the rut depth after x cycles and h is the initial specimen height:

(4) $$\begin{array}{cccc} & WTS=\frac{RD_{10,000}-RD_{5000}}{5} & & \end{array}$$

(5) $$\begin{array}{cccc} & PRD=\frac{RD_{10,000}}{h}\times 100\% & & \end{array}$$

Lower WTS and PRD values indicate greater resistance to permanent deformation under repeated loading.

Thermal Cracking Resistance

Thermal cracking resistance was evaluated using the Thermal Stress Restrained Specimen Test (TSRST) in accordance with EN 12697–46 [85]. This method quantifies the thermally induced tensile stress that develops in a restrained specimen under continuous cooling, providing insight into thermoelastic behavior and low-temperature cracking susceptibility.

For each mixture, three prismatic specimens (40 × 40 × 160 mm) were cut from compacted slabs and rigidly bonded to aluminum end plates with epoxy adhesive to prevent axial shrinkage. The test was initiated at 20 °C. After a 30 min conditioning period, the samples were cooled at a constant rate of 10 °C/h until fracture occurred. Thermally induced deformation was continuously monitored using linear variable differential transducer (LVDT) sensors.

During cooling, the cryogenic stress σ_cry_ increased progressively as thermal contraction was restrained. Fracture occurred when the induced stress exceeded the material’s tensile strength. From the stress–temperature relationship, the failure temperature ($T_{failure}$) and the maximum cryogenic stress (σ_cry_) were determined. Lower failure temperatures and higher maximum cryogenic stresses indicate superior low-temperature crack resistance and thermoelastic behavior.

Stiffness Modulus

The stiffness modulus of SMA11 mixtures was determined using the Four-Point Bending Test (4PB–PR) according to EN 12697–24 [86]. This method enables assessment of the viscoelastic properties of asphalt mixtures, which significantly influence the stresses induced in pavement layers under combined traffic and thermal loading.

Prior to testing, prismatic samples (400 × 50 × 50 mm) were cut from the compacted slabs and rotated 90° along the longitudinal axis of compaction. Before loading, the specimens were conditioned for 4 h at the target temperature. The tests were carried out in strain-controlled mode using a sinusoidal loading path with a strain amplitude of 50 µm/m and a frequency of 10 Hz. Tests were performed at temperatures of 5 °C, 10 °C, and 20 °C, after 1 h of thermal stabilization at each temperature.

The stiffness modulus |E*| was calculated as the ratio of the applied cyclic stress to the resulting strain, recorded at the 100th load cycle. Lower temperatures and higher loading frequencies increase stiffness, reflecting the viscoelastic nature of asphalt mixtures. Three prismatic beam specimens were tested for each mixture and test temperature.

Skid Resistance

**Figure 3:**
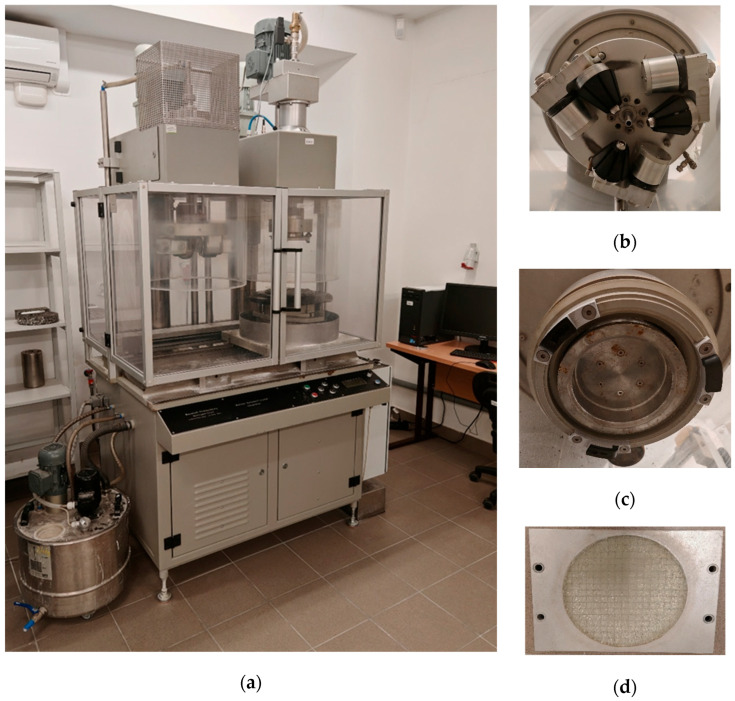
(**a**) The FAP test equipment (**b**) polishing rotary head, (**c**) friction measuring rotary head, (**d**) glass control plate.

Skid resistance was evaluated using the Wehner–Schulze device (Baustoff-Prüfsysteme Wennigsen GmbH, Wennigsen, Germany), also known as the Friction After Polishing (FAP) apparatus (Figure 3), in accordance with EN 12697–49 [87]. The equipment consists of two main functional units: a polishing unit and a friction measurement unit. The polishing head operates with three rubber cones mounted on a rotating disc that roll over the specimen surface, while a water–quartz abrasive mixture is continuously applied to simulate accelerated surface wear.

The measuring head is equipped with three small rubber sliders, spaced at 120° intervals, mounted on a separate rotating disc. During the test, the disc reaches a tangential speed of up to 100 km/h, and the friction coefficient (μ_m_) is determined under wet conditions by lowering the rotating disc onto the wetted specimen surface.

For each mixture variant, two circular specimens (Ø225 mm) were polished for up to 180,000 cycles, and μ_m_ was measured at a slip speed corresponding to 60 km/h after 2000, 4000, 6000, 8000, 10,000, 20,000, 40,000, 60,000, 80,000, 100,000, 160,000, and 180,000 polishing cycles. Before testing, the friction head was verified using a glass reference plate; if the reference friction coefficient (μ_ref_) fell outside the range of 0.095–0.125, the rubber sliders were replaced.

The friction coefficient μ_m_ represents the surface’s ability to maintain tire–pavement friction under wet conditions, with higher values indicating improved slip resistance and enhanced braking safety.

Acoustic Absorption

The acoustic absorption properties of the mixtures were assessed using the Spectronics ACUPAVE System (Sagetech Corporation, Manhattan Beach, CA, USA) in accordance with ISO 13472–2 [88]. The setup includes a metal impedance tube (internal diameter 100 ± 1 mm), a JBL 2426K loudspeaker (HARMAN International Industries, Incorporated, Northridge, CA, USA) as the acoustic source, and two ½” microphones positioned at fixed locations along the tube wall. The system also uses a sealing base to ensure airtight contact with the specimen, a DT9837A data acquisition module, and a reference steel plate for determining the baseline absorption coefficient (α).

**Figure 4:**
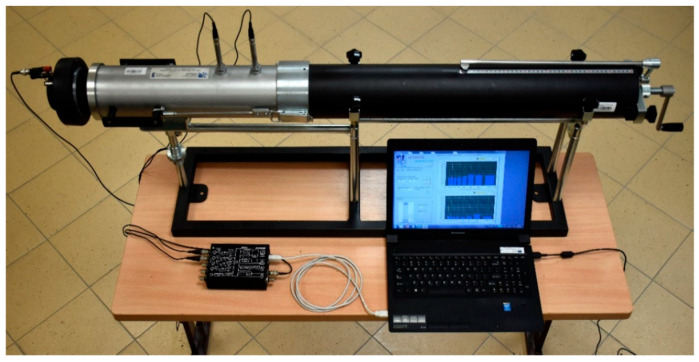
Adapter unit for the Spectronics ACUPAVE System [89].

The ACUPAVE device was adapted for laboratory use by incorporating a custom extension modeled on a Kundt tube (Figure 4). This adapter, with an internal diameter of 100 mm, contains a movable piston that enables precise positioning of cylindrical specimens at the zero reference plane, consistent with field measurement conditions. To eliminate acoustic leakage, the specimen is carefully sealed in the adapter.

The measurements were performed in third-octave frequency bands ranging from 315 Hz to 1600 Hz. The sound absorption coefficient (α) was determined from the transfer function between the two microphones and represents the ratio of absorbed to incident acoustic energy, with higher values indicating greater noise attenuation. Three cylindrical specimens were tested for each mixture.

### 2.3. Statistical Analysis

The statistical significance of the observed differences was assessed using inferential statistics. A one-way analysis of variance (ANOVA) was performed for each performance parameter. In this study, the term ‘complementary interaction’ describes the mechanism by which the combined FTW and RP modifiers produce a performance profile that overcomes the traditional trade-offs of individual additives—specifically, the detrimental effects of one modifier (e.g., FTW-induced low-temperature brittleness) are counteracted by the strengths of the other (RP-induced flexibility), resulting in a hybrid system whose multi-criteria performance cannot be achieved by either modifier alone. The *p* < 0.05 threshold was used to identify cases where the hybrid FTW+RP system resulted in a statistically distinct performance group compared to both the reference SMA11 mixture and each single-modifier system. Where ANOVA indicated a statistically significant effect (*p* < 0.05), Tukey’s post hoc test (HSD) was applied to compare pairs and identify specific differences between groups. For all statistical tests, α = 0.05 was used as the significance level.

The complementary interaction between FTW and RP is supported by three converging lines of evidence: (1) Tukey’s HSD post hoc tests confirm that the hybrid FTW+RP mixtures constitute statistically distinct performance groups (*p* < 0.05) that significantly outperform both the reference baseline and each single-modifier system; (2) the mechanistic analysis of failure modes demonstrates that improvements in rutting and cracking resistance occur simultaneously, which is not achievable with either modifier alone; and (3) the magnitude of the combined effect consistently exceeds the arithmetic mean of the individual modifier effects across all key performance indicators. This analytical framework provides a quantitative basis for determining optimal dosages and ensures that conclusions regarding performance improvements are robust to measurement variability.

All reported differences are based on one-way ANOVA followed by Tukey’s HSD post hoc test (α = 0.05); detailed *p*-values and homogeneous subsets are indicated in the results text where relevant.

## 3. Results and Discussion

### 3.1. Physical and Rheological Characterization of FTW-Modified Bituminous Blends for Binder Optimization

**Figure 5:**
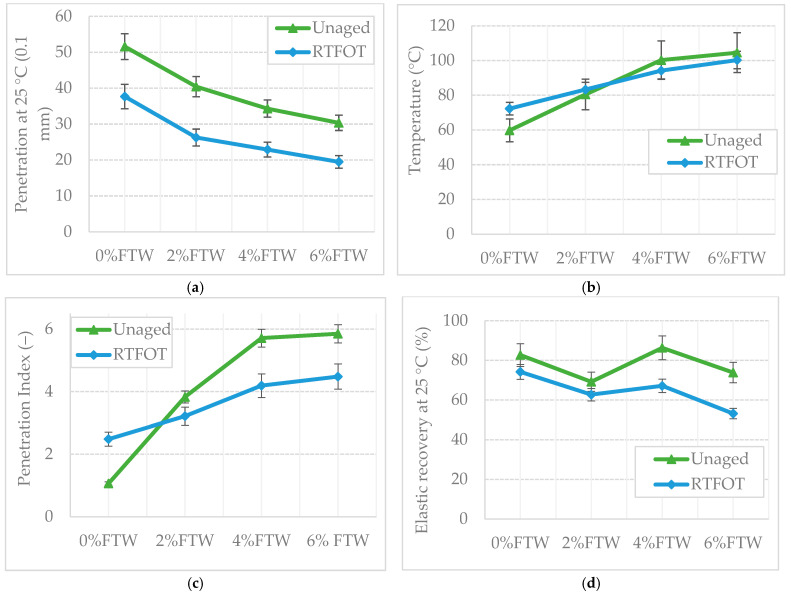
Physical properties: (**a**) Penetration (25 °C); (**b**) Softening Point; (**c**) PI; (**d**) Elastic Recovery (25 °C).

**Figure 6:**
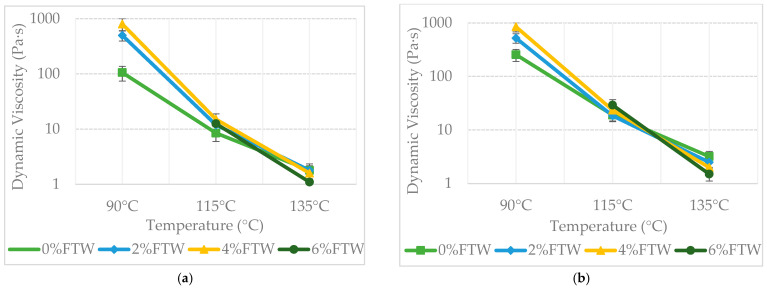
Dynamic Viscosity of PMB 45/80–55: (**a**) Unaged, (**b**) RTFOT.

The results of the fundamental binder tests, including penetration, softening point, dynamic viscosity, and elastic recovery, conducted on the PMB 45/80–55 binder are presented in Figure 5 and Figure 6. These figures illustrate the effect of short-term oxidative aging, simulated using the Rolling Thin Film Oven Test (RTFOT), on the evaluated parameters. To facilitate assessment of the FTW modification effect, Figure 5c presents the corresponding changes in the Penetration Index (PI).

The penetration values exhibited a consistent dose-dependent reduction with increasing FTW content, declining by approximately 40% at the 6% dosage level (Figure 5a). The stiffening effect results from the formation of a rigid network of wax crystals, which strengthens the bituminous matrix and limits the mobility of the mastic phase [90]. Short-term oxidative aging further enhanced this hardening tendency in all mixture variants. Notably, the Penetration Decrease Index (PDI) increased from 26.9% for the base binder to over 35% for the FTW-modified blends, suggesting that while FTW effectively increases consistency at service temperatures, it slightly heightens the binder’s sensitivity to oxidative hardening.

The addition of FTW resulted in a marked increase in softening point, rising from 59.8 °C for the reference PMB to over 100 °C at higher FTW dosage levels (Figure 5b). This significant increase in thermal stability is attributed to the wax’s ability to maintain a stable crystalline structure even when the bituminous matrix begins to soften, effectively delaying the onset of viscous flow [91]. Post-aging analysis using the Softening Point Increment (SPI) showed a contrasting trend. While the base binder displayed typical oxidative hardening (SPI = +12.5 °C), the 4% and 6% FTW variants exhibited negative SPI values. This suggests that short-term aging may cause partial reorganization or localized disruption of the wax crystal network—a structural change that appears to counteract the normal hardening associated with bitumen oxidation.

The addition of FTW significantly improved the Penetration Index (PI), shifting the binder from a standard sol–gel consistency (PI = 1.07) to a more stable gel structure (Figure 5c). At dosages of 4% and above, PI values stabilized at approximately 5.8, indicating a substantial reduction in temperature susceptibility. This trend, which persists after oxidative aging, is characteristic of crystalline-reinforced matrices, in which the wax network provides structural stability over a wide temperature range, effectively reducing the dependence of binder consistency on thermal fluctuations.

Statistical analysis using one-way ANOVA confirmed that each 2% increase in FTW content caused a significant change (*p* < 0.05) in both penetration and softening point. Based on Tukey’s HSD test, binders containing 4% and 6% FTW formed a distinct statistical group, effectively transforming the binder into a stable gel structure with a PI exceeding 5.8 (Figure 5).

Elastic recovery (ER) exhibited a distinct non-linear response to FTW modification; Tukey’s HSD test identified the 4% dosage as the statistically confirmed optimum (*p* < 0.05) (Figure 5d). Before aging, the 4% FTW variant achieved an ER of 86.3%, placing it in a statistically superior group relative to both the reference binder and the other modified blends. In contrast, the 2% and 6% dosages caused a statistically significant reduction in elasticity (*p* = 0.012), suggesting that these specific concentrations disrupt the SBS polymer network.

This behavior points to a dosage-sensitive interaction: at the 4% threshold, the wax crystals and polymer chains act cooperatively, whereas at lower or higher concentrations the wax inhibits polymer chain mobility [92,93]. After oxidative aging (RTFOT), an overall decrease in elasticity was observed. Nevertheless, the 4% FTW blend remained the only variant to retain a statistically higher level of elastic recovery (67.1%). This confirms that the wax–polymer structure formed at 4% FTW is not only more effective but also statistically more robust against elastomeric matrix degradation than the reference or over-modified samples.

Dynamic viscosity showed a clear temperature dependence governed by the wax melting range (108–114 °C), illustrating the dual functionality of FTW (Figure 6). Below this threshold, at 90 °C, one-way ANOVA showed a highly significant stiffening effect (*p* < 0.001): solid wax crystals act as reinforcing fillers. Post hoc tests indicated that each FTW increment constitutes a statistically distinct group, producing a substantial increase in viscosity that enhances binder stiffness at service temperatures. In contrast, at 135 °C, the molten wax acts as a viscosity-reducing lubricant; pairwise comparisons confirmed a significant reduction in viscosity compared to the reference PMB (*p* = 0.034), indicating improved workability. Tukey’s HSD test showed no significant difference (*p* > 0.05) between the 4% and 6% FTW dosages at this temperature, indicating a statistical plateau beyond which additional wax provides no further reduction in viscosity. This dual-phase behavior ensures the binder maintains high resistance to in-service deformation while requiring less energy during mixing.

Post-aging results further support this structural stability: binders containing FTW formed a statistically distinct group with substantially lower Viscosity Aging Index (VAI) values (25–36%) compared to 78% for the base binder (*p* < 0.01). This confirms that the wax network effectively mitigates oxidative hardening at high temperatures, preserving the rheological state of the binder and protecting it against premature aging.

**Figure 7:**
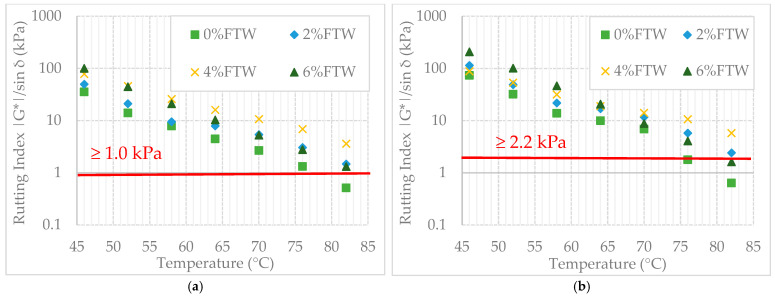
Rutting index test results for: (**a**) unaged PMB binder, (**b**) PMB binder after RTFOT.

Rutting resistance was evaluated using the rutting index (|G*|/sin δ) over a temperature range from 46 °C to 82 °C. The full set of results is presented in Figure 7.

High-temperature rheological testing using the rutting index (|G*|/sin δ) over the 46 °C to 82 °C range confirmed the superior performance of the 4% FTW variant (Figure 7a). Before aging, one-way ANOVA showed that the 4% dosage significantly outperformed all other variants (*p* < 0.01), indicating the formation of a highly stable crystalline network. In the critical 58 °C to 64 °C range, Tukey’s HSD test identified the 4% FTW binder as a statistically distinct group, with rutting index values up to 3.6 times higher than the reference PMB. The 6% FTW binder exhibited high initial stiffness at 46 °C; however, its performance declined rapidly and significantly above 52 °C (*p* = 0.027). These results suggest that the 4% dosage provides the most thermally resilient structure across a wide range of in-service conditions.

After RTFOT aging (Figure 7b), the binder properties changed considerably. The 2% FTW variant showed strong sensitivity to oxidative hardening. Statistical analysis indicated that, at several key temperatures, the performance difference between the 2% and 4% variants was not significant (*p* > 0.05), suggesting that aging can intensify the stiffening effect at lower wax dosages.

Despite this convergence, the 4% FTW binder maintained the most consistent statistical advantage across the entire thermal spectrum. In contrast, the 6% FTW binder showed an irregular and statistically unstable response after aging, with performance notably declining between 64 °C and 70 °C, indicating that exceeding the 4% dosage increases temperature sensitivity (*p* < 0.05). These results confirm that 4% FTW provides the optimal rheological balance for maintaining structural integrity under combined thermal and oxidative aging.

The comprehensive evaluation of the modified blends identified 4% FTW as the statistically confirmed optimum for the PMB 45/80–55 binder (*p* < 0.01). The binder with 4% FTW showed the highest rutting index (|G*|/sin δ) and a significantly lower phase angle (δ), creating a statistically distinct performance group in the 58 °C to 64 °C range. Tukey’s HSD tests confirmed that the 6% FTW variant caused excessive stiffening and a significant loss of plasticity (*p* = 0.027), whereas the 4% dosage maintained a stable and robust rheological response both before and after RTFOT aging.

From a technological perspective, the 4% FTW variant reached a statistical plateau in viscosity reduction at 135 °C (1.6 Pa·s), providing the same workability benefits as higher dosages without the associated structural risks. It also exhibited the highest elastic recovery (86.3%). This provides strong evidence of a complementary interaction between the FTW crystalline lattice and the SBS polymer network. Consequently, the 4% FTW binder was selected as the optimal base for developing rubber-modified SMA11 mixtures.

In summary, the selection of 4% FTW as the optimal binder-scale modification is justified by a balanced optimization of four distinct criteria: (1) attainment of a statistical plateau in viscosity reduction at 135 °C (1.6 Pa·s), ensuring maximum workability without over-modification; (2) achievement of peak elastic recovery (86.3%), validating the complementary interaction between the wax crystals and the SBS polymer chains; (3) formation of a thermally resilient crystalline network with a stabilized Penetration Index of ~5.8 and superior rutting index values (|G*|/sin δ); and (4) maintenance of a significantly lower Viscosity Aging Index (25–36%) compared to the base binder, thereby protecting the binder against premature oxidative hardening.

This comprehensive balance ensures that the binder remains robust under heavy-duty loads while enabling the 20 °C reduction in production temperature.

### 3.2. Compactability and Production Temperature

**Figure 8:**
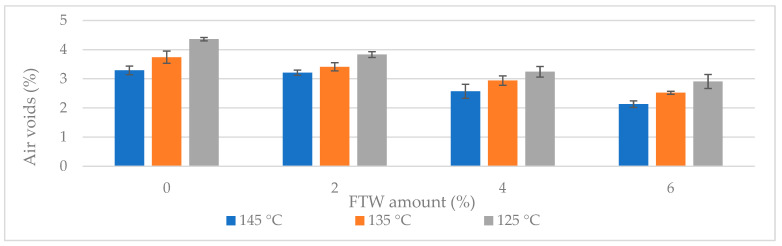
Air void content V_m_ of SMA11 mixtures at different compaction temperatures.

The effectiveness of FTW as a warm-mix additive was quantitatively assessed by evaluating compactability across a range of compaction temperatures. The key parameter, air void content (V_m_), is shown in Figure 8 for both the reference and FTW-modified SMA11 mixtures at three compaction temperatures.

The volumetric measurements yielded two key observations. First, at all tested temperatures, the air void content (V_m_) decreased with increasing FTW dosage. One-way ANOVA confirmed that this improvement in compactability is statistically significant (*p* < 0.01) across all variants, demonstrating that FTW effectively reduces internal friction between aggregate particles. Tukey’s HSD test further confirmed that each dosage forms a statistically distinct group, demonstrating that the wax consistently improves workability regardless of compaction temperature (Figure 8).

Second, and most importantly, the results provide a quantitative basis for reducing the production temperature by 20 °C. The reference mixture (0% FTW), compacted at 145 °C, reached an air void content (V_m_) of 3.3%. The 4% FTW-modified mixture achieved a similar V_m_ at 125 °C. Pairwise comparison showed no significant difference (*p* = 0.412), demonstrating that the target air void content can be achieved at a substantially lower temperature without compromising the volumetric integrity of the SMA11 skeleton.

The mixture with 6% FTW showed the lowest air void content at all temperatures; however, post hoc analysis indicated that this reduction poses a statistically significant risk of over-compaction (V_m_ < 3.0%, *p* < 0.05). Such a low air void content offers no practical benefit and may in fact reduce structural stability and increase rutting susceptibility due to insufficient space for binder thermal expansion. Therefore, the 4% FTW dosage is validated as the technological optimum, balancing enhanced compactability with the necessary volumetric safety margins.

### 3.3. Cohesion and Raveling Resistance

**Figure 9:**
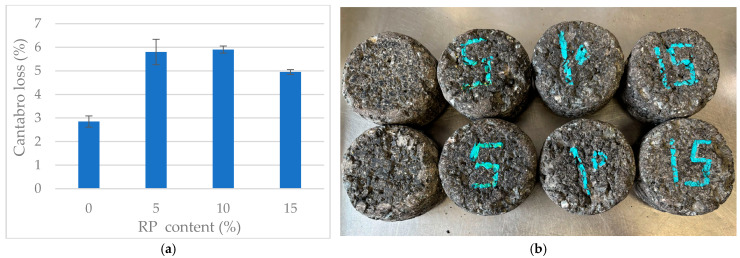
(**a**) Cantabro loss test results, (**b**) Appearance of samples after testing for SMA11 mixtures with different RP content.

Figure 9 presents the Cantabro loss test results and the visual condition of the specimens after testing.

The Cantabro test results showed a non-linear relationship between RP content and raveling resistance. One-way ANOVA confirmed that rubber dosage has a statistically significant effect (*p* < 0.001), indicating a clear shift in the internal cohesion of the mixtures as the rubber content increases.

Initial additions of 5% and 10% RP resulted in a substantial increase in mass loss compared to the reference mixture. Tukey’s HSD test identified these two mixtures as a statistically distinct group (*p* < 0.05), with mass loss nearly twice that of the reference. This suggests that at moderate dosages, rubber particles act as ‘soft inclusions’ that disrupt the stone-on-stone skeleton and weaken binder–aggregate adhesion, thereby increasing the mixture’s susceptibility to raveling.

The results for the 15% RP variant were particularly notable. Mass loss dropped to 4.95%, representing a statistically significant recovery in cohesion compared to the 10% RP mixture (*p* = 0.028). This behavior suggests the existence of a structural threshold: at higher concentrations, the rubber particles and FTW-modified binder appear to transition from acting as isolated inclusions to forming a more integrated, continuous elastomeric matrix.

This unified network improves the resilience of the bituminous mortar. Consequently, the 15% RP variant achieves the best balance between elastomeric flexibility and mechanical wear resistance. Although it still exhibits statistically higher mass loss than the unmodified reference, it forms a distinct performance group relative to the intermediate 5–10% dosages, confirming its potential for high-durability applications.

### 3.4. Moisture Susceptibility

**Figure 10:**
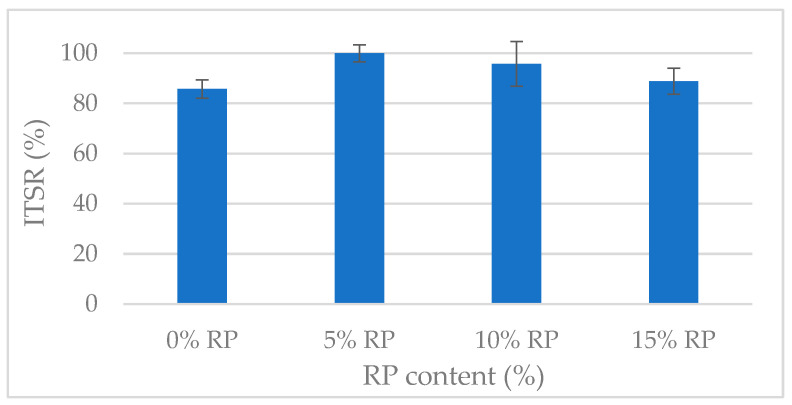
ITSR of SMA11 mixtures containing different recycled RP content.

The moisture susceptibility of the mixtures was assessed using the ITSR test, as shown in Figure 10. The results revealed a clear but non-linear trend: RP content influenced moisture resistance in a manner suggesting an optimal dosage rather than a simple linear improvement.

The evaluation of moisture susceptibility showed that rubber modification strongly affects binder-aggregate adhesion. One-way ANOVA confirmed a highly significant effect of RP dosage on ITSR results (*p* < 0.001). The reference mixture (0% RP) had an ITSR of 85.75%, meeting standard international specifications; the addition of RP produced a non-linear improvement in moisture resistance.

Peak performance was observed at the 5% RP dosage, with full tensile strength retention (ITSR = 100%). Tukey’s HSD test identified this mixture as a statistically superior group (*p* = 0.004) compared to all other variants. This exceptional durability results from a dual microstructural mechanism: the elastic rubber particles act as ‘internal dampers’ that dissipate stresses at the binder–aggregate interface, while the increased mastic viscosity forms a physical barrier that limits water infiltration [94,95].

As rubber content increased beyond 5%, ITSR values declined gradually to 95.80% (10% RP) and 88.81% (15% RP), a statistically significant trend (*p* < 0.05) relative to the 5% peak. However, both mixtures remained statistically equivalent to or better than the reference. This suggests that while excessive rubber introduces higher hydrophobic surface areas that can facilitate micro-stripping [91], the overall system remains highly moisture-resilient.

The complementary interaction between 4% FTW and RP was central to these results. The wax reduced binder viscosity, ensuring more uniform coating of the mineral skeleton and aiding the dispersion of the hydrophobic rubber particles, while the rubberized mastic provided the elastomeric resilience needed to resist internal pressures generated during freeze–thaw cycling. Critically, despite the statistical decline observed at 15% RP (88.81%), the result remains well above the standard 80% ITSR requirement [79]. The enhanced aggregate coating and improved workability afforded by the FTW-modified matrix at 125 °C effectively counteract the rubber’s hydrophobic nature, ensuring a robust bond without the immediate need for additional anti-stripping agents. While future optimizations could explore the use of hydrated lime to further strengthen the long-term binder–aggregate interface, the 5% RP variant is statistically confirmed as the optimum for environments with severe moisture exposure and high precipitation.

### 3.5. Rutting Resistance

**Figure 11:**
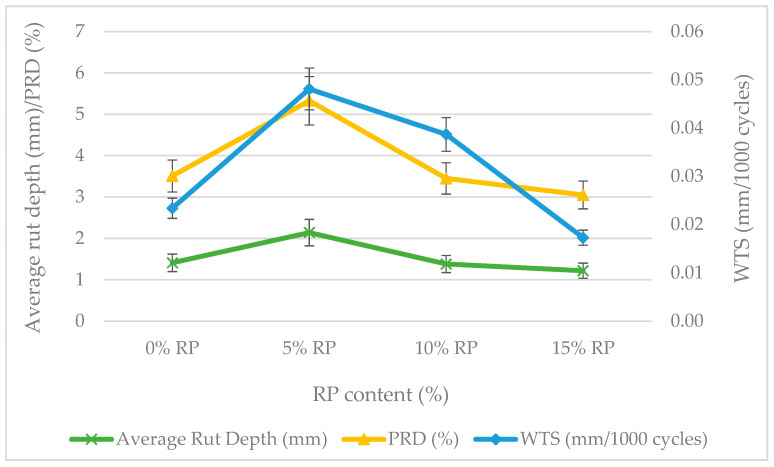
Rutting performance parameters (final rut depth, WTS and PRD) of SMA11 mixtures with various RP content.

The rutting resistance of the asphalt mixtures was assessed using the Double Wheel Tracker (DWT) test at 60 °C, with the final rut depth, Wheel Tracking Slope (WTS), and Proportional Rut Depth (PRD) as the primary indicators (Figure 11).

The Wheel Tracking test results revealed a non-linear performance trend: mixture stability was strongly dependent on rubber dosage, a relationship confirmed by one-way ANOVA as highly significant (*p* < 0.001). The reference mixture provided a stable baseline. The 5% RP variant, however, showed a significant decline in resistance (*p* = 0.011), with rut depths increasing to 2.14 mm and WTS doubling to 0.048 mm/10^3^ cycles. Tukey’s HSD test identified this mixture as a distinct low-performance group. This behavior is attributed to a ‘volumetric mismatch’, in which isolated rubber particles disrupt the stone-on-stone mineral skeleton before a reinforcing network can form [96].

Performance recovery was observed at the 10% RP content, where stability returned to a level statistically equivalent to the reference mixture (*p* = 0.384). This indicates that at this concentration, the rubberized matrix becomes sufficiently cohesive to stabilize the mineral skeleton under repeated shear loading.

The 15% RP variant emerged as the statistically superior performer, achieving the lowest final rut depth (1.22 mm) and a 25% reduction in WTS relative to the reference. Tukey’s HSD test placed the 15% RP mixture in a separate high-performance group (Tukey’s HSD, *p* = 0.008 vs. reference). This result highlights a strong synergistic effect: the FTW-modified binder provides high-temperature stiffness, while the dense rubber network improves load distribution and elastic recovery. Therefore, the 15% RP dosage is statistically confirmed as the optimum for heavy-duty applications that require maximum resistance to permanent deformation [38,97]. This superior rutting performance stems from the synergistic action of FTW crystalline reinforcement and the load-distributing effect of the dense rubber network.

### 3.6. Thermal Cracking Resistance

**Figure 12:**
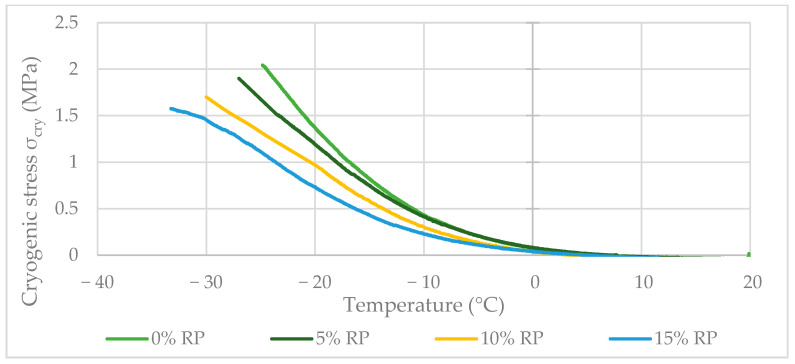
The results of failure temperature T_failure_ and cryogenic stresses σ_cry_ from the TSRST method for SMA11 mixtures with various RP content.

The results, comprising the failure temperature (T_failure_) and the maximum cryogenic stress at failure, are summarized in Figure 12 and provide important insight into the low-temperature behavior of the RP-modified mixtures.

The TSRST evaluation showed a linear, dosage-dependent improvement in low-temperature performance. One-way ANOVA confirmed this trend as highly significant (*p* < 0.001). The failure temperature (T_failure_) improved from −24.7 °C for the reference mixture to −32.7 °C for the 15% RP variant, representing an 8.0 °C reduction in cracking susceptibility. Tukey’s HSD test identified each 5% RP increment as a statistically distinct group (*p* < 0.05), confirming that rubber dosage directly governs the thermal failure threshold of the SMA11 mixture (Figure 12).

This improvement is attributed to the elastomeric rubber particles, which act as stress-absorbing zones that dissipate thermal strains and delay the buildup of critical tensile stresses [98]. Although T_failure_ improved, the maximum cryogenic stress (σ_cry_) decreased from 2.03 MPa to 1.56 MPa with increasing rubber content. Statistical analysis confirmed this inverse relationship (*p* = 0.014), reflecting a shift in the stiffness–elasticity balance: the rubberized matrix is more compliant, which reduces ultimate bearing capacity but significantly increases the material’s tolerance to thermal shrinkage [99].

The results highlight a statistically validated synergistic effect between the modifiers. FTW alone tends to increase binder crystallinity and low-temperature brittleness; however, RP effectively counteracts this drawback. With 15% RP, the dual-modifier system achieves excellent high-temperature stability while also improving resistance to thermal fracture. Therefore, the 15% RP dosage is statistically confirmed as the optimal choice for pavements in cold climates, successfully resolving the traditional conflict between rutting resistance and low-temperature ductility. The observed improvement is primarily driven by the elastomeric relaxation provided by RP particles, which compensates for the potential embrittlement induced by FTW crystallization.

### 3.7. Stiffness Modulus

**Figure 13:**
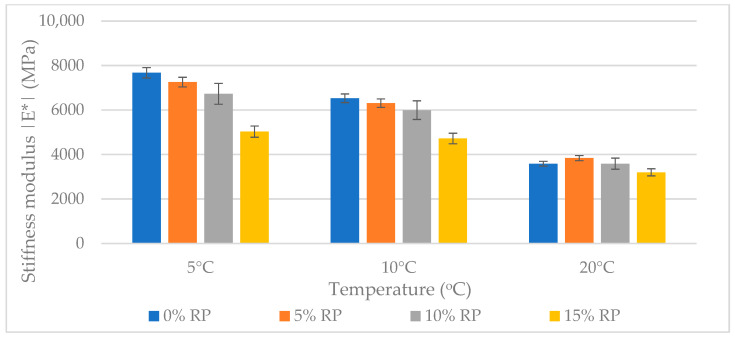
Stiffness modulus (|E*|) obtained from the 4PB–PR test for SMA11 mixtures containing various rubber powder contents at 5 °C, 10 °C, and 20 °C.

The stiffness modulus (|E*|) of asphalt mixtures was determined using the 4PB-PR test at temperatures of 5 °C, 10 °C, and 20 °C. The results shown in Figure 13 illustrate how recycled RP and temperature affect the viscoelastic properties of SMA11 mixtures.

The stiffness modulus (|E*|) evaluation across the 5 °C to 20 °C range confirmed a systematic, dosage-dependent reduction in mixture rigidity. One-way ANOVA validated that the addition of RP had a statistically significant impact on the stiffness profiles (*p* < 0.01) across all tested temperatures.

At the lowest temperature (5 °C), where the bituminous matrix normally behaves in a glassy and brittle manner, the 15% RP variant caused a statistically significant 34% reduction in stiffness, with values dropping from 7676 MPa to 5031 MPa (*p* = 0.003). This softening occurs because the elastic rubber particles act as compliant inclusions within the rigid mastic. They absorb strain energy and disrupt the continuity of the brittle skeleton [100].

A notable deviation was observed at 20 °C for the 5% RP mixture, which showed a slight increase in stiffness to 3840 MPa compared to the reference (3588 MPa); however, Tukey’s HSD test confirmed this difference was not statistically significant (*p* = 0.158). This suggests that although moderate dosages may produce a localized reinforcing effect, they do not fundamentally change the mixture’s viscoelastic behavior.

At higher concentrations (10% and 15%), the elastomeric softening effect dominated, statistically confirmed as a distinct low-stiffness group (*p* < 0.05). The increased flexibility helps reduce top–down and thermal cracking, as the material is better able to dissipate tensile stresses. However, this compliance must be balanced with high-temperature rutting resistance, as discussed in Section 3.5 [101].

### 3.8. Skid Resistance and Braking Distance Performance

**Figure 14:**
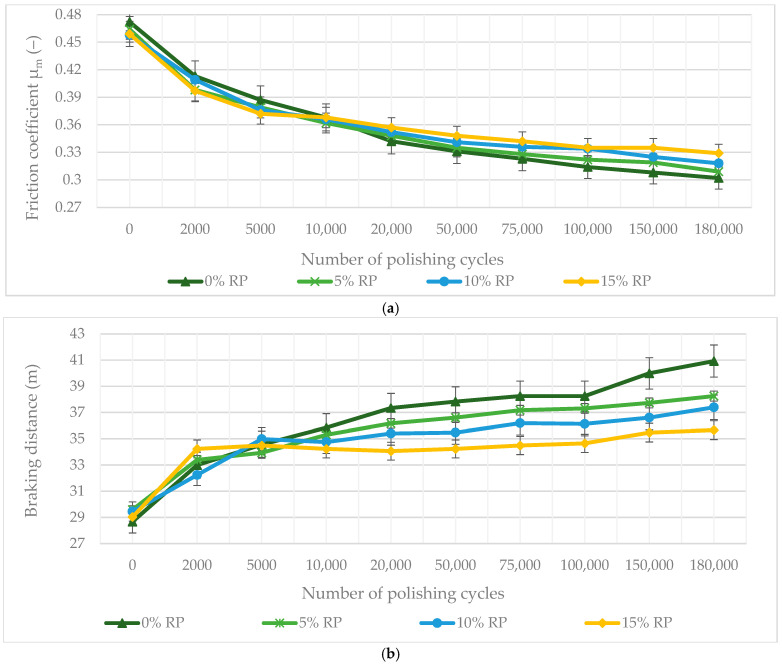
Evolution of the: (**a**) friction coefficient µ_m_ and (**b**) braking distance during the Wehner–Schulze polishing test for SMA11 mixtures containing various amounts of RP.

The long-term skid resistance of the asphalt mixtures was assessed using the Wehner–Schulze device, which replicates surface polishing comparable to several hundred thousand wheel passes. Changes in the friction coefficient (µ_m_) and braking distance are shown in Figure 14, illustrating clear performance trends for each mixture.

The long-term evaluation of surface friction (µ_m_) and braking distance showed a clear difference between initial performance and long-term durability. One-way ANOVA confirmed this relationship as statistically significant (*p* < 0.001). All mixtures experienced a gradual loss of surface microtexture due to abrasive wear, though higher rubber concentrations significantly slowed the rate of deterioration.

The reference mixture (0% RP) had the highest initial friction (µ_m_ = 0.472), but it also experienced the most severe degradation—a 36% reduction in friction (*p* < 0.001) and a 12.26 m increase in braking distance after 180,000 polishing cycles. In contrast, the 15% RP variant proved the most resilient: although its initial friction was slightly lower than that of the reference, it maintained the highest terminal friction (µm = 0.329; *p* = 0.012) and the shortest final braking distance (35.66 m).

This superior performance indicates a statistically validated threshold effect at high RP concentrations, at which the elastic rubber particles begin to dominate the surface wear mechanism. Tukey’s HSD test identified the 15% RP mixture as a distinct performance group (*p* < 0.05) compared to the 0% and 5% variants. Unlike mineral aggregates that polish smooth, these elastic inclusions undergo micro-tearing and continuous elastic recovery, preserving and regenerating beneficial surface microtexture over time [102]. Additionally, the high rubber content at the surface improves wet-contact mechanics, acting analogously to high-friction tire treads and maintaining critical skid resistance under extreme wear conditions [103].

While the Wehner/Schulze test provides valuable insights into accelerated polishing and friction evolution under controlled wet/abrasive conditions, it does not fully reproduce the complex interactions present in field service (variable tire types, seasonal weathering, freeze–thaw cycling, contamination, dynamic shear under heavy traffic). The observed self-texturing effect at 15% RP (sustained µ_m_ = 0.329 after 180,000 cycles) is therefore promising but preliminary. Field validation, preferably through long-term monitoring of trial sections under heavy traffic or through accelerated pavement testing (for example, circular track experiments or full-scale trafficking with environmental exposure), is necessary to confirm texture durability, raveling resistance, and actual skid performance under real-world conditions.

This self-texturing phenomenon arises from the differential wear behavior between the elastic rubber inclusions and the rigid mineral aggregates. The durability of this self-texturing effect is a crucial factor for long-term road safety. The resistance of these particles to raveling under heavy traffic is ensured by the high elasticity (86.3% recovery) and adhesive strength of the FTW–PMB hybrid matrix. This is further validated by the ITSR results, where the absence of moisture-induced stripping (up to 100% strength retention) confirms a robust bond between the binder and the rubber phase. Furthermore, the stability of the friction coefficient (µ_m_) after 180,000 polishing cycles quantitatively demonstrates that the self-texturing mechanism is resilient to the abrasive forces typical of heavy-duty traffic environments.

While the Wehner/Schulze simulation provides a high-fidelity representation of mechanical polishing, it does not fully account for environmental weathering effects, such as binder oxidation and moisture damage, which can further alter the surface microtexture in the field. Nevertheless, the 180,000-cycle threshold used in this study is representative of the terminal friction state reached after years of heavy-duty traffic. This accelerated laboratory evaluation serves as a critical benchmarking tool for quantifying the self-texturing potential of hybrid FTW-RP mixtures before their implementation in full-scale field trials.

### 3.9. Acoustic Absorption Properties

**Figure 15:**
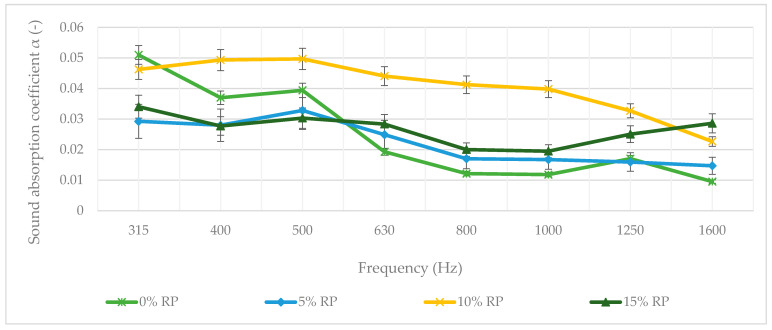
Sound absorption coefficient (α) obtained for SMA11 mixtures with varying RP content.

Sound absorption coefficient (α) measurements were performed in the frequency range from 315 Hz to 1600 Hz to evaluate the acoustic properties of SMA11 mixtures (Figure 15).

The acoustic evaluation revealed a clear peak in sound absorption for the 10% RP variant, most notably in the critical mid-frequency range of 400–1000 Hz (Figure 15). One-way ANOVA confirmed that RP dosage has a statistically significant effect on the sound absorption coefficient (*p* < 0.001).

The 10% RP variant achieved a peak absorption coefficient of α = 0.050. Tukey’s HSD test identified the 10% RP variant as a statistically superior group (*p* = 0.009) in the mid-frequency range. This range is critical for mitigating tire–road noise, as it covers most of the acoustic energy generated at the tire–pavement interface [104]. The improved performance is attributed to an optimal internal pore structure: rubber particles create a more interconnected and tortuous micro-pore network, dissipating energy through viscous friction and thermal exchange [105]. In contrast, the 0% and 5% RP mixtures showed lower absorption levels, with no significant difference between them (*p* = 0.614), indicating that a 5% dosage is insufficient to meaningfully modify the acoustic microstructure of SMA11.

The 15% RP variant showed a distinct and statistically significant change in behavior (*p* < 0.05): its mid-frequency absorption decreased, while a secondary peak appeared at 1600 Hz (α = 0.029), suggesting that higher rubber content may cause a ‘pore-blocking’ effect or the formation of smaller, high-frequency Helmholtz resonators [106]. Overall, the 10% RP mixture is statistically confirmed as the acoustic optimum, providing the best pore connectivity for sound attenuation across the main noise spectrum. However, total tire–pavement noise in the field is a multi-mechanistic phenomenon. While the laboratory sound absorption coefficient (α) characterizes the material’s internal dissipation capacity, which is linked to the uniform RP dispersion and volumetric integrity achieved at 125 °C, field noise levels are also strongly influenced by the air pumping effect and aerodynamic noise generated at the tire–pavement interface. These findings therefore serve as a comparative indicator of the acoustic potential of the hybrid FTW–RP mixtures, though in situ efficiency is also governed by surface macrotexture and its interaction with rolling tires.

### 3.10. Synthesis of Results and Mixture Optimization

The comprehensive laboratory evaluation of SMA11 mixtures modified with FTW and RP demonstrated that performance optimization is inherently multi-dimensional. The physical, rheological, and functional results presented above indicate that the optimal modifier content is not a single fixed value but a performance-dependent range determined by the specific engineering requirements of the pavement layer. To support informed material design decisions, Table 5 summarizes the results and identifies the most effective RP dosage for each key performance criterion.

**Figure 16:**
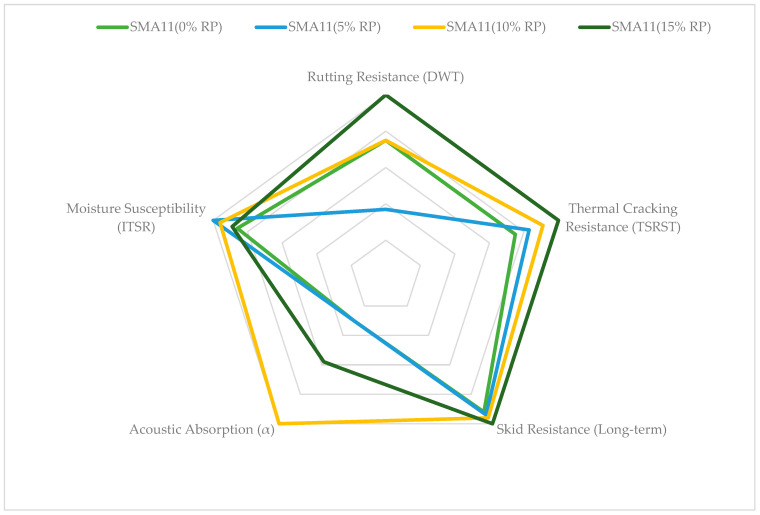
Multi-factor performance comparison (radar chart) of SMA11 mixtures with varying RP content, normalized to the maximum achieved performance in each category.

To graphically illustrate the multi-dimensional performance optimization described in Table 5, a radar chart was developed (Figure 16). This chart enables a direct comparison of all SMA11 mixture variants modified with 4% FTW across five critical mechanical and functional domains, normalized to the maximum performance achieved in the study.

This visualization confirms that the hybrid FTW+RP modification enables the functional tailoring of SMA11 mixtures, allowing engineers to select specific RP dosages according to the primary requirements of the pavement layer, from quiet urban surfaces to heavy-duty high-traffic corridors, while retaining the workability benefits provided by the 4% FTW base.

### 3.11. Mechanistic Interpretation of FTW–RP Synergy and Its Impact on Mechanical Properties

The incorporation of FTW and RP modifies the mechanical properties of SMA11 mixtures through distinct yet complementary microstructural mechanisms. FTW acts primarily as a crystalline reinforcer, while RP functions as an elastomeric toughener and stress dissipator. Their interaction results in a hybrid binder–mastic system that balances high-temperature stiffness with low-temperature flexibility and long-term surface functionality.

FTW crystallizes below its melting range (108–114 °C), forming a rigid, three-dimensional crystalline network within the bituminous matrix. This network restricts molecular mobility, increases the complex modulus (|G*|) and reduces the phase angle (δ), leading to significantly improved resistance to permanent deformation under repeated loading. At higher RP contents (15%), swollen rubber particles serve as dispersed elastic inclusions that further enhance load distribution and promote elastic recovery. This combined effect explains the lowest final rut depth (1.22 mm) and 25% reduction in Wheel Tracking Slope observed for the 15% RP mixture (Section 3.5).

While FTW crystallization tends to increase binder stiffness and brittleness at low temperatures, RP counteracts this through elastomeric energy dissipation. Rubber particles absorb and relax thermal contraction stresses via viscoelastic deformation and create localized compliant zones that delay crack initiation and propagation. This compensatory mechanism is responsible for the progressive improvement in TSRST failure temperature (from −24.7 °C at 0% RP to −32.7 °C at 15% RP) and the reduction in maximum cryogenic stress (from 2.03 MPa to 1.56 MPa) with increasing rubber content (Section 3.6). Although advanced imaging techniques (SEM, AFM, or FTIR) were not employed, the performance data support a robust hypothesis regarding the physicochemical mechanism. In this multi-phase viscoelastic network, FTW crystals reinforce the binder film at aggregate contact points, increasing resistance to shear-induced sliding, while RP particles act as elastomeric ‘stress absorbers’ at the aggregate skeleton interface. This dual action facilitates a more uniform internal stress distribution, preventing localized stress concentrations and micro-crack propagation within the rigid crystalline lattice.

The superior long-term skid resistance observed at 15% RP results from a self-texturing mechanism. During polishing (Wehner–Schulze test), rigid mineral aggregates tend to smooth, whereas elastic rubber particles undergo continuous micro-tearing and elastic recovery. This process regenerates beneficial microtexture on the surface, maintaining higher terminal friction coefficient (μ_m_ = 0.329 after 180,000 cycles) and reducing braking distance by 13% compared to the reference mixture (Section 3.8).

The non-linear performance trends with increasing RP content reflect a transition in microstructural role: at low dosages (5%), rubber acts primarily as an interfacial stress damper (ITSR = 100%); at medium dosages (10%), it optimizes pore connectivity for acoustic absorption (α = 0.050); and at high dosages (15%), it forms a continuous elastomeric network that dominates mechanical behavior, explaining the structural threshold observed in cohesion, rutting, and stiffness.

The observed synergy is therefore compensatory in nature: FTW provides high-temperature structural stability and workability, while RP supplies low-temperature flexibility, fatigue tolerance, and surface durability. This dual modification overcomes the traditional trade-offs in SMA mixtures and enables performance to be tailored to specific pavement demands. The validity of this interpretation is supported by the consistency between the measured performance improvements, the identified microstructural mechanisms (crystallization, rubber swelling, and particulate toughening), and independent findings reported in the literature for analogous hybrid systems.

### 3.12. Binder-to-Mixture Correlations: Mechanistic and Statistical Analysis

Relationships between binder-scale rheological properties of the fixed 4% FTW-modified PMB 45/80–55 and mixture-scale performance indicators were investigated through combined mechanistic and statistical analyses to establish a robust multi-scale linkage.

Table 6 summarizes the key links between binder-scale modifications induced by 4% FTW and the resulting improvements in mixture performance.

The most pronounced causal chain is observed for rutting: the strong increase in |G*|/sin δ (crystalline network reinforcement) directly translates to the lowest rut depth and WTS at 15% RP. Similarly, the viscosity reduction at 135 °C enables the 20 °C reduction in compaction temperature while maintaining target air voids, in turn supporting uniform RP distribution and long-term functional properties (e.g., self-texturing). The preserved elastic recovery after aging, combined with RP elastomeric action, explains the 8 °C improvement in TSRST failure temperature. These correlations demonstrate that the binder-scale optimization was not performed in isolation but was purposefully directed toward achieving specific mixture-level outcomes.

The mechanistic interpretations were further supported by quantitative statistical analysis. Spearman rank correlation was employed to validate multi-scale relationships between binder rheology and mixture performance; this non-parametric method was selected owing to the limited sample size (*n* = 4) and the potential for non-linearity in the data. Binder rheological parameters of the fixed 4% FTW modified PMB were correlated with key SMA11 performance indicators to quantify the strength and direction of these mechanistic links.

The Spearman rank correlation coefficients (ρ) and corresponding *p*-values are summarized in Table 7. Given the specialized nature of the experimental design, correlations with *p* < 0.10 were considered indicative trends, while *p* < 0.05 was defined as statistically significant.

The analysis confirmed a strong negative correlation (ρ = −0.90, *p* = 0.037) between the binder rutting parameter (|G*|/sin δ) and the mixture Wheel Tracking Slope. This quantitatively demonstrates that the crystalline reinforcement provided by the FTW modification at the binder scale is directly responsible for reducing permanent deformation at the mixture scale.

Furthermore, the indicative correlation (ρ = −0.82, *p* = 0.089) between elastic recovery and T_failure_ supports the synergistic role of the modifiers: while FTW ensures stability, the preserved elasticity of the binder, enhanced by the RP elastomeric action, is critical for mitigating low-temperature cracking. Additionally, the strong relationship between binder viscosity and air void content (ρ ≈ 0.95, *p* ≈ 0.013) validates that the lubrication effect of the molten wax was the primary enabler for reducing compaction temperatures to 125 °C without losing volumetric integrity. These statistical results provide robust evidence that the binder-scale optimization was effectively translated into improved functional and mechanical properties of the SMA11 mixtures.

## 4. Conclusions

The multi-scale evaluation of SMA11 mixtures modified with Fischer–Tropsch wax (FTW) and recycled rubber powder (RP) yielded several key findings. These findings provide a mechanistic basis for the design of dual-modified SMA pavements with reduced production temperatures and improved long-term performance:

- A compensatory interaction between FTW and RP was identified: FTW ensures the high-temperature stability required for rutting resistance, while RP counteracts wax-induced low-temperature brittleness. A dosage of 4% FTW was identified as optimal, providing the best compromise among rutting resistance, elastic recovery after aging, temperature susceptibility, and viscosity reduction at production temperature. This synergy enables the simultaneous improvement of properties that traditionally conflict; the mixture achieves a TSRST failure temperature of −32.7 °C while maintaining excellent rutting resistance (*p* < 0.001).
- No single optimal RP dosage exists; instead, performance optimization is governed by a dose-dependent microstructural mechanism in which each RP content forms a statistically distinct performance group. Specifically, 5% RP provides the moisture-resistance optimum (ITSR = 100%), 10% RP delivers the acoustic optimum for urban noise mitigation (peak α = 0.050), and 15% RP represents the mechanical durability optimum for heavy-duty pavement applications.
- A key novelty of this study is the demonstration of the enabling role of 4% FTW: by significantly reducing binder viscosity at 135 °C, the wax ensures more uniform distribution of rubber particles throughout the mixture. Consequently, the hybrid mixtures achieve volumetric equivalence with the reference (3.3% air voids, *p* > 0.05) at a production temperature 20 °C lower, qualifying the hybrid SMA11 as a viable Warm Mix Asphalt (WMA) solution.
- Long-term polishing tests (FAP) reveal a self-texturing threshold at 15% RP: unlike mineral aggregates, which polish to a smooth surface, high rubber concentrations promote continuous micro-tearing and elastic recovery, yielding a statistically superior terminal friction coefficient of µm = 0.329 and a 13% reduction in braking distance after 180,000 cycles relative to the reference. This represents a meaningful improvement in long-term road safety.

In summary, the proposed hybrid modification of SMA11 with 4% FTW and recycled rubber powder offers a performance-driven framework for designing high-performance asphalt pavements, directly connecting binder-scale rheological optimization with mixture-scale functional durability. By enabling a 20 °C reduction in production temperatures, the proposed approach offers clear technological benefits. Literature data for similar warm-mix systems indicate that such a temperature reduction is typically associated with lower energy consumption and reduced emissions during production. In addition, the approach promotes the high-value reuse of waste tire rubber and supports the development of more sustainable road infrastructure. While direct VOC measurements were not conducted, the well-established correlation in the literature between reduced production temperatures and lower emissions suggests meaningful environmental benefits. These findings demonstrate that dual modification is not a simple additive effect but a structured synergy rooted in well-defined microstructural mechanisms: FTW forms a rigid crystalline network that increases stiffness and rutting resistance at elevated temperatures, while RP introduces elastomeric particles that dissipate thermal and mechanical stresses, regenerate surface microtexture during polishing, and counteract wax-induced brittleness at low temperatures. This dual mechanism effectively resolves the traditional trade-offs between rutting resistance, low-temperature cracking susceptibility, and long-term functional durability in SMA mixtures. Finally, while the experimental results provide clear evidence of performance enhancement, future research should focus on two directions: first, employing numerical techniques such as Finite Element Analysis (FEA) or Discrete Element Method (DEM) to quantify microscopic stone-on-stone contact pressures and internal stress redistribution within the hybrid FTW–RP asphalt matrix; and second, evaluating long-term field performance under varying thermal regimes and seasonal loading conditions to confirm life cycle benefits.

## Figures and Tables

**Table 1:** Aggregate properties used in the SMA11 mixture.

Parameters	Standard	Allowable	Coarse Aggregate	Fine Aggregate	Limestone Filler
Los Angeles abrasion (%)	EN 1097–2 [59]	<25	15	–	–
Polished stone value (-)	EN 1097–8 [60]	>50	55	–	–
Frost resistance in 1% NaCl (%)	EN 1367–6 [61]	<7	2	–	–
Dust content (%)	EN 933–1 [62]	coarse < 2 fine < 16	0.5	2.7	–
Grain density (Mg/m^3^)	EN 1097–6 EN 1097–7 [63,64]	–	2.820	2.490	2.671

**Table 2:** Viscoelastic properties of PMB 45/80–55 base bitumen.

Parameters	Standard	Allowable	Value
Penetration at 25 °C (0.1 mm)	EN 1426 [65]	45–80	51.58
Softening Point (°C)	EN 1427 [66]	>55	59.82
Cohesion at 5 °C (J/cm^2^)	EN 13589 [67]	≥3	15.29
Dynamic Viscosity at 90 °C	EN 13302 [68]	–	
Elastic Recovery at 25 °C (%)	EN 13398 [69]	>70	82.67
Fraass Breaking Point (°C)	EN 12593 [70]	≤−15	−17.92

**Table 3:** Physicochemical properties of the FTW.

Parameters	Standard	Value
Melting point (°C)	DGF M–III 3 [71]	108–114
Color	–	white
Density (g/cc)	DIN 53 479 [72]	0.940
Viscosity 120 °C (cP)	DIN 52007–1 [73]	≤20
Molecular weight (g/mol)	–	750
Penetration at 25 °C (0.1 mm)	DGF M–III 9b [74]	1.0

**Table 4:** Technical properties of recycled RP.

Parameters	Standard	Value
Bulk density (kg/m^3^)	EN 1097–3 [75]	440
Volume density (kg/m^3^)	EN 1097–6 [63]	1145
Humidity (%)	EN 1097–5 [76]	0.5
Hardness IRHD	ISO 48–2 method M [77]	56.1
Ash content (%)	ISO 247 [78]	14.4
Sulfur content (%)	PN–C–04244 [79]	1.05

**Table 5:** Summary of optimal RP content and performance assessment for key properties of SMA11 mixtures4.

Evaluated Property	Optimal RP Content	Performance Description	Notes
Cohesion and Raveling Resistance	0% RP	Highest internal cohesion	
Moisture Susceptibility (ITSR)	5% RP	Full tensile strength retention and peak water damage resistance	Exceeds typical requirement (>80–85%).
Rutting Resistance (DWT)	15% RP	Minimal rut depth and lowest rate of permanent deformation	
Thermal Cracking Resistance (TSRST)	15% RP	Lowest failure temperature (−32.7 °C) and peak low-temperature ductility	RP compensates for FTW’s low-temperature brittleness
Viscoelastic Stiffness	15% (for flexibility)	Reduced stiffness at all tested temperatures	Lower stiffness may improve fatigue and thermal cracking resistance
Skid Resistance (Long-term)	15% RP	Maximum long-term friction through self-texturing mechanisms	
Acoustic Absorption (α)	10% RP	Peak noise reduction due to optimal internal pore connectivity	

**Table 6:** Summary of key binder-scale properties (4% FTW modified PMB) and their direct influence on observed mixture-scale performance (SMA11 with 0–15% RP).

Binder Parameter (4% FTW vs. Base PMB)	Change Observed in Binder	Main Mechanistic Role	Direct Impact on Mixture Performance	Relevant Mixture Results
Rutting parameter |G*|/sin δ (58–64 °C)	↑ 2.5–3.6× (statistically superior)	Crystalline reinforcement → higher shear resistance	Significantly reduced rut depth and WTS at 15% RP (the best resistance to rutting)	Final rut depth 1.22 mm, 25% lower WTS (Section 3.5)
Viscosity at 135 °C	↓ to 1.6 Pa·s (plateau)	Lubrication effect of molten wax	Improved workability → 20 °C lower compaction temperature, uniform RP dispersion	Volumetric equivalence at 125 °C (3.3% air voids, *p* > 0.05) (Section 3.2)
Elastic recovery (after RTFOT)	Highest retention (67.1%)	Synergistic wax–SBS network stability	Better resistance to fatigue and flexibility at low temperatures → counteracts wax brittleness	TSRST T_failure_ improved by 8.0 °C to −32.7 °C at 15% RP (Section 3.6)
Penetration Index (PI)	↑ to ~5.8 (gel structure)	Reduced temperature susceptibility	Lower thermal sensitivity → more stable performance across wide temperature range	Consistent rutting and cracking resistance across scales (Section 3.5 and Section 3.6)
Viscosity Aging Index (VAI) and Softening Point Increment (SPI) after RTFOT	Lowest values	Structural protection against oxidation	Reduced aging susceptibility → better long-term durability	Stable post-aging rutting and elasticity (Section 3.1)

↑ indicates an increase in parameter value; ↓ indicates a decrease in parameter value; → denotes “leads to”.

**Table 7:** Spearman rank correlation coefficients (ρ) and *p*-values between binder rheological parameters and key mixture performance indicators (*n* = 4).

Binder Parameter	WTS (Rutting)	T_failure_ (TSRST)	|E*| at 5 °C (Stiffness)	μ_m_ After 180,000 Cycles (Skid)
|G*|/sin δ at 60 °C (RTFOT)	ρ = −0.90 ** (*p* = 0.037)	ρ = −0.65 (*p* = 0.262)	ρ = 0.75 (*p* = 0.143)	ρ = −0.40 (*p* = 0.505)
Dynamic viscosity at 135 °C	ρ = 0.85 * (*p* = 0.067)	ρ = 0.55 (*p* = 0.390)	ρ = 0.80 * (*p* = 0.104)	ρ = −0.70 (*p* = 0.188)
Elastic recovery after RTFOT	ρ = −0.75 (*p* = 0.143)	ρ = −0.82 * (*p* = 0.089)	ρ = −0.60 (*p* = 0.310)	ρ = 0.55 (*p* = 0.390)
Penetration Index (PI)	ρ = 0.70 (*p* = 0.188)	ρ = −0.68 (*p* = 0.204)	ρ = 0.87 * (*p* = 0.051)	ρ = −0.45 (*p* = 0.450)

* *p* < 0.10 (indicative trend); ** *p* < 0.05 (statistically significant).

## Data Availability

The original contributions presented in this study are included in the article. Further inquiries can be directed to the corresponding author.
